# Multi-Granularity Domain Adversarial Learning for Cross-Domain Tea Classification Using Electronic Nose Signals

**DOI:** 10.3390/foods15081376

**Published:** 2026-04-15

**Authors:** Xiaoran Wang, Yu Gu

**Affiliations:** 1 College of Information Science and Technology, Beijing University of Chemical Technology, Beijing 100029, China; 2021400222@buct.edu.cn; 2 School of Biomedical Engineering, Capital Medical University, Beijing 100069, China

**Keywords:** electronic nose, tea classification, domain adaptation, multi-granularity feature learning, domain adversarial learning

## Abstract

Rapid and reliable tea classification is valuable for routine product screening, yet conventional sensory or physicochemical methods are subjective or time-consuming. Electronic nose (E-nose) sensing provides a fast alternative, but performance often degrades under domain shifts caused by different tea types, commercial categories, or acquisition conditions. This study proposes MGDA-Net, a multi-granularity domain adversarial network for cross-domain tea classification using E-nose time-series signals. MGDA-Net learns local temporal dynamics via a CNN branch and global contextual dependencies via a self-attention branch, and fuses them through an adaptive gating module. A branch-level adversarial alignment strategy is introduced to reduce source–target discrepancy at both local and global feature levels. A three-stage training procedure, consisting of source pretraining, adversarial alignment, and target fine-tuning, enables knowledge transfer from a labeled green tea source-domain to two target tasks. Experiments on oolong tea commercial-category classification (6 classes) and jasmine tea retail price-level classification (8 classes) show that MGDA-Net achieves mean accuracies of 99.31 ± 0.69% and 99.38 ± 0.51% over 10 independent runs, substantially outperforming all compared baseline methods. Ablation studies, feature-space analyses, and label-efficiency experiments further confirm the contribution of each component and show that MGDA-Net maintains mean accuracies above 87% when only 40% of the target-domain labels are used for fine-tuning. These findings suggest that MGDA-Net is a promising approach for cross-domain tea classification using E-nose data.

## 1. Introduction

Tea is one of the most widely consumed beverages worldwide and also a high-value agricultural commodity with substantial economic, cultural, and commercial significance [1,2]. According to official statistics from the National Bureau of Statistics of China, national tea production reached 3.74 million tons in 2024, representing a 5.5% year-on-year increase [3]. At the global level, tea production generates more than USD 18 billion annually and supports the livelihoods of approximately 13 million people [4]. With the continuous expansion and diversification of the tea market, reliable tea classification and product differentiation have become increasingly important for product grading, commercial management, market supervision, and consumer protection. In practice, premium teas may be misclassified, mislabeled, or mixed with lower-value products, while adjacent commercial categories often exhibit only subtle sensory differences [5,6,7]. These issues create a strong demand for rapid, objective, and scalable methods for tea classification under realistic industrial conditions.

Conventional tea evaluation still relies mainly on sensory assessment and physicochemical analysis [8,9]. Sensory evaluation remains an important reference approach in routine grading, but it depends heavily on trained panelists and is therefore inherently subjective, labor-intensive, and vulnerable to inter-observer variability [10,11]. Physicochemical methods, such as chromatographic and spectrometric analyses of catechins, amino acids, and volatile compounds, can provide more objective evidence, but they usually require expensive instrumentation, complex sample preparation, destructive testing, and time-consuming analytical procedures [12,13]. Several rapid and non-destructive alternatives, including near-infrared spectroscopy and hyperspectral imaging, have also been explored for tea analysis, yet their performance may still be sensitive to measurement geometry, instrument calibration, and environmental fluctuations [14,15,16]. Against this background, the electronic nose (E-nose), which captures aroma-response patterns through sensor arrays, has attracted increasing attention because of its rapid response, non-destructive measurement, operational simplicity, and potential for low-cost on-site screening [17,18]. Representative studies have already demonstrated the applicability of E-nose methods in tea-related tasks. For example, Kang et al. [19] combined an E-nose with an adaptive pooling attention mechanism to discriminate teas from different picking periods, Yu and Gu [20] developed a CNN–SVM framework for fine-grained green tea classification with geographical indication, and Chang and Lu [21] proposed RLCSA-Net for E-nose-based tea identification. These studies collectively indicate that E-nose signals contain informative patterns for tea category discrimination, commercial-label-related classification, and origin identification.

Despite these advances, most reported E-nose models are still developed and evaluated under single-domain or closed-set conditions, where the training and test samples are collected under highly similar acquisition settings [22,23]. In practical deployment, however, E-nose measurements may vary across acquisition days, batches, tea types, grading criteria, and ambient conditions, leading to domain shifts that can substantially degrade model performance when a trained classifier is transferred to a new target task [24,25,26]. This challenge becomes more pronounced in cross-category tea classification, where the source and target tasks may differ not only in data distribution but also in class definition. Domain adaptation has been widely studied to mitigate cross-domain distribution discrepancy in machine learning [27,28]. In E-nose research, existing adaptation methods have mainly focused on sensor drift correction or single-condition transfer [29,30,31,32,33]. However, E-nose signals are multichannel time-series in which domain discrepancy may appear simultaneously in local transient dynamics (e.g., rise and recovery patterns) and global contextual structure (e.g., long-range temporal trends and inter-sensor dependencies) [34,35]. Methods that align only a single representation space may therefore overlook complementary cross-domain cues and be insufficient for fine-grained classification scenarios [36,37,38,39]. Consequently, an important remaining research gap is the lack of domain adaptation frameworks that explicitly model and align E-nose features at multiple granularities for robust cross-domain tea classification.

To address this gap, this study proposes a multi-granularity domain adversarial network (MGDA-Net) for cross-domain tea classification using E-nose time-series signals. Green tea is used as the labeled source-domain, and knowledge is transferred to two heterogeneous target tasks, namely oolong tea commercial-category classification and jasmine tea retail price-level classification. Accordingly, the present study focuses on cross-domain classification of heterogeneous commercial label sets rather than on independently validated sensory or chemical quality grading. MGDA-Net combines a convolutional neural network (CNN) branch to learn local temporal dynamics, a self-attention branch to model global contextual dependencies, an adaptive fusion module to integrate the two branches, and a branch-level adversarial alignment strategy within a progressive three-stage transfer framework. The main contributions of this work are summarized as follows:

1. A multi-granularity feature learning framework is proposed to jointly capture local temporal patterns and global contextual dependencies from multichannel E-nose signals. The framework integrates a CNN branch for local dynamic feature extraction, a self-attention branch for global contextual modeling, and an adaptive fusion module with learnable gating for dynamic integration of heterogeneous branch features.
2. A branch-level adversarial domain adaptation strategy is developed to reduce source–target discrepancy simultaneously at local and global feature levels. Combined with a three-stage transfer procedure, this design improves the transferability and robustness of the learned representations in cross-domain tea classification.
3. Comprehensive evaluations are conducted on two heterogeneous target tasks with different classification criteria and difficulty levels. Comparisons with five target-only baselines and three representative domain adaptation baselines, together with ablation studies, multi-seed robustness analysis, feature-level analyses, and label-efficiency experiments, validate the effectiveness and stability of MGDA-Net.

## 2. Materials and Methods

### 2.1. Tea Samples

Three tea datasets were used in this study to evaluate the cross-domain classification performance of the proposed model, as summarized in Table 1.

The source-domain dataset was adopted from our group’s previous study [20]. It consisted of 12 varieties of Chinese green tea (Camellia sinensis) collected in Beijing, China, in 2020. The samples were sourced from major tea-producing provinces, including Anhui, Zhejiang, Sichuan, Guizhou, Henan, Guangxi, Hunan, and Shaanxi. The 12 varieties comprised six Maofeng-type teas (Huangshan Maofeng, Yandang Maofeng, Emei Maofeng, Lanxi Maofeng, Qishan Maofeng, and Meitan Maofeng) and six Maojian-type teas (Xinyang Maojian, Guilin Maojian, Duyun Maojian, Guzhang Maojian, Queshe Maojian, and Ziyang Maojian). In total, 1440 green tea samples were used as the source-domain dataset.

Target-domain 1 consisted of six classes of oolong tea collected in Xiamen, China, in 2022. All samples were provided by the Xiamen Institute of Quality Inspection and Testing. The six classes included four non-branded yancha (rock tea) samples, namely Kuidou Shuixian (Premium, hereafter O1), Kuidou Shuixian (Grade I, hereafter O2), Foguoyan Shuixian (A++, hereafter O3), and Foguoyan Shuixian (A, hereafter O4), as well as two branded products, Xupinhao Shuixian (hereafter O5) and Xupinhao Rougui (hereafter O6). The first two Shuixian classes originated from Kuidou Village, Yongchun County, Fujian Province, while the latter two Shuixian classes originated from the Foguoyan area of Wuyi Mountain, Fujian Province. Each class contained 60 samples, yielding 360 samples in total.

Target-domain 2 consisted of eight classes of jasmine tea collected in Beijing, China, in 2025. All samples were produced by Wu Yutai, a well-known Chinese tea brand based in Beijing. The eight classes were defined by their retail price levels, ranging from CNY 50 to CNY 400 per 500 g at increments of CNY 50, representing different commercial price tiers. Each class contained 60 samples, resulting in 480 samples in total.

Green tea was selected as the source-domain because it shares a common botanical origin and partially overlapping volatile organic compound profiles with both oolong tea and jasmine tea, while exhibiting processing-induced differences [40,41,42]. This design enables the evaluation of cross-category domain adaptation, where knowledge learned from one tea category is transferred to classify chemically related categories under distribution shifts.

### 2.2. E-Nose System and Data Acquisition

All E-nose measurements were performed using a PEN3 portable E-nose system (Airsense Analytics GmbH, Schwerin, Germany), which is equipped with an array of 10 metal oxide semiconductor (MOS) gas sensors. Each sensor exhibits selective sensitivity to different groups of volatile compounds. The source-domain data adopted from our previous study were acquired using the same PEN3 instrument type and a consistent acquisition protocol as those used for the target-domain datasets.

For each measurement, approximately 4.0 g of tea sample was placed in a sealed 50-mL glass vial and equilibrated at room temperature for 3 min to allow headspace volatiles to accumulate. All measurements were conducted under controlled laboratory conditions at 25 ± 1 °C and 40 ± 2% relative humidity. Filtered ambient air was used as the carrier gas at a constant flow rate of 600 mL/min. Each acquisition cycle consisted of two stages. In the cleaning stage, filtered air was passed through the sensor chamber for 100 s to restore the sensor baseline. In the subsequent sampling stage, the headspace gas from the sample vial was introduced into the sensor chamber for 120 s. The sampling interval was 1 s, resulting in 120 time points for each of the 10 sensors. Therefore, each measurement was represented as a 10 × 120 response matrix describing the temporal evolution of the sensor array.

Based on the above acquisition procedure, three datasets were constructed for the cross-domain experiments. The source-domain dataset consisted of 1440 measurements from 12 green tea varieties, collected over 12 days, with 10 samples per variety measured each day (one measurement per sample). The oolong tea dataset contained 360 samples from 6 classes (60 samples per class), and the jasmine tea dataset contained 480 samples from 8 classes (60 samples per class).

For each target-domain dataset, measurements were collected over 10 consecutive days, with 6 measurements acquired per class per day. To simulate a realistic cross-session deployment scenario, a day-wise split was adopted. Specifically, the data collected on days 1–8 were used as the target-domain training portion, whereas the data collected on days 9–10 were reserved as the target-domain test portion. Accordingly, the oolong tea dataset was divided into 288 training measurements and 72 test measurements, and the jasmine tea dataset was divided into 384 training measurements and 96 test measurements. This temporal split ensured that the final evaluation was performed on measurements obtained in independent acquisition sessions rather than on randomly mixed data from the same session.

For the oolong tea dataset, each of the 60 samples per class was treated as a physically independent unit. The four non-branded yancha classes were provided as individually packaged quality inspection samples by the Xiamen Institute of Quality Inspection and Testing, with each measured sample originating from a distinct production lot. The two branded products (Xupinhao Shuixian and Xupinhao Rougui) were sourced from separate retail packages. No sample was measured more than once. The day-wise train–test split therefore ensures that training and test partitions reflect separate acquisition sessions with no overlapping measured samples.

For the jasmine tea dataset, each of the 60 samples per class was prepared from a separately opened retail package of Wu Yutai jasmine tea at the corresponding price level. This package-level sampling introduced within-class variability beyond repeated measurement of a single package. Each sample was measured exactly once. As with the oolong tea dataset, the day-wise temporal split ensures that training and test samples were collected in independent acquisition sessions.

In the cross-domain experiments, the green tea dataset served as the labeled source-domain, while the oolong tea and jasmine tea datasets were treated as two independent target domains. During Stage 2 domain adversarial training, the target-domain training samples participated in feature alignment without using their class labels. During Stage 3 fine-tuning, the same target-domain training samples were used with their class labels for supervised classifier adaptation. The held-out test set was used exclusively for final evaluation and was not involved in any stage of model training or model selection. Accordingly, the proposed setting should be interpreted as a supervised three-stage transfer framework rather than an unsupervised target-domain adaptation setting.

### 2.3. Evaluation Metrics

To comprehensively assess the classification performance of the proposed model, five evaluation metrics were adopted in this study. Overall accuracy (OA) measured the proportion of correctly classified samples among all test samples, providing the most intuitive indication of model performance. It is defined as:

(1) $$OA=\frac{\sum_{i=1}^{N}1\left({\hat{y}}_{i}=y_{i}\right)}{N}$$

where *N* denotes the total number of test samples, $y_{i}$ and ${\hat{y}}_{i}$ are the true and predicted labels of the *i*-th sample, and 1(·) is the indicator function.

For each class *c*, precision $P_{c}$ and recall $R_{c}$ were defined as:

(2) $$P_{c}=\frac{TP_{c}}{TP_{c}+FP_{c}},\;R_{c}=\frac{TP_{c}}{TP_{c}+FN_{c}}$$

where $TP_{c}$, $FP_{c}$, and $FN_{c}$ denote the numbers of true positives, false positives, and false negatives for class *c*, respectively. Macro-averaged precision ($P_{macro}$) and macro-averaged recall ($R_{macro}$) were computed by averaging per-class values equally across all *C* classes:

(3) $$P_{macro}=\frac{1}{C}\sum_{c=1}^{C}P_{c},\;R_{macro}=\frac{1}{C}\sum_{c=1}^{C}R_{c}$$

Macro-averaged precision reflected the model’s ability to avoid false positive errors across all classes, while macro-averaged recall captured its ability to correctly identify samples from each class.

The macro-averaged F1-score (F1-macro) was obtained by first computing the harmonic mean of $P_{c}$ and $R_{c}$ for each class and then averaging across all classes

(4) $$F1_{macro}=\frac{1}{C}\sum_{c=1}^{C}\frac{2P_{c}R_{c}}{P_{c}+R_{c}}$$

By treating all classes equally regardless of sample size, this metric provided a balanced evaluation of the trade-off between precision and recall, making it particularly suitable for multi-class classification tasks.

Cohen’s kappa coefficient (κ) quantified the degree of agreement between predicted and true labels beyond what would be expected by chance, offering a more rigorous evaluation than accuracy alone. It is defined as:

(5) $$\kappa =\frac{p_{0}-p_{e}}{1-p_{e}}$$

where $p_{0}$ is the observed agreement (equivalent to OA) and $p_{e}$ is the expected agreement under random classification.

## 3. Proposed Method

### 3.1. Overview of MGDA-Net

**Figure 1:**
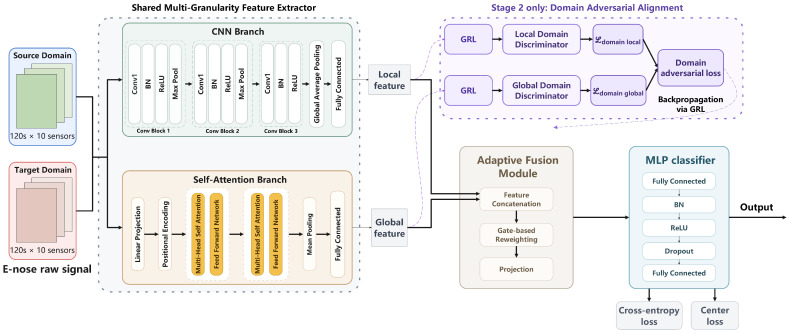
Overall architecture of the proposed MGDA-Net.

The proposed multi-granularity domain adversarial network (MGDA-Net) is designed to transfer discriminative knowledge learned from a labeled source-domain to a target domain for E-nose-based tea classification. The overall architecture, illustrated in Figure 1, consists of four main components: (1) a multi-granularity feature extractor comprising a CNN branch and a self-attention branch, (2) an adaptive fusion module that dynamically combines local and global features, (3) a multi-granularity domain adversarial module with two independent discriminators operating at different feature granularities, and (4) a multilayer perceptron (MLP) classifier with center loss regularization.

Given an input E-nose measurement $X\in R^{C\times T}$, where C=10 denotes the number of sensor channels and T=120 denotes the number of time steps, the network first applies instance normalization across each channel to reduce sensor-level distributional variability. The normalized input is then processed in parallel by the CNN branch and the self-attention branch to extract local and global feature representations, respectively. These two representations are combined through the adaptive fusion module to produce a fused feature vector, which is subsequently fed into the classifier for category prediction. During domain adversarial training, the local and global features are independently passed through gradient reversal layers and domain discriminators to align the source- and target-domain distributions at multiple granularities.

### 3.2. Multi-Granularity Feature Extraction

#### 3.2.1. CNN Branch for Local Feature Extraction

The CNN branch is designed to capture local temporal patterns and short-range dependencies in the E-nose response signals. It consists of three successive convolutional blocks followed by a fully connected layer. Each convolutional block contains a one-dimensional convolution, batch normalization, ReLU activation, and max pooling. The first two blocks employ a kernel size of 5 with 32 and 64 filters, respectively, while the third block uses a kernel size of 3 with 128 filters. After the third convolutional block, adaptive average pooling is applied to compress the temporal dimension to a single value, resulting in a 128-dimensional vector. This vector is then projected through a fully connected layer with batch normalization and ReLU activation to produce the local feature representation $f_{cnn}\in R^{d}$, where d=128. The local feature extraction process can be summarized as:

(6) $$f_{cnn}=\mathrm{FC}\left(\mathrm{AdaptiveAvgPool}\left({\mathrm{ConvBlock}}_{3}\left({\mathrm{ConvBlock}}_{2}\left({\mathrm{ConvBlock}}_{1}\left(X\right)\right)\right)\right)\right)$$

The relatively small kernel sizes and hierarchical pooling operations enable the CNN branch to focus on localized sensor response patterns and fine-grained temporal variations, which are essential for distinguishing tea samples with subtle differences in volatile compound profiles.

#### 3.2.2. Self-Attention Branch for Global Feature Extraction

The self-attention branch is designed to model long-range temporal dependencies and capture global contextual information across the entire measurement sequence. The input $X\in R^{C\times T}$ is first transposed to $R^{T\times C}$ and projected to a higher-dimensional space through a linear layer with ReLU activation, yielding $H_{0}\in R^{T\times d_{m}}$, where $d_{m}=64$ is the model dimension.

Sinusoidal positional encodings are then added to preserve temporal order information

(7) $$PE_{\left(pos,2i\right)}=\sin\left(\frac{pos}{{10,000}^{2i/d_{m}}}\right),\;PE_{\left(pos,2i+1\right)}=\cos\left(\frac{pos}{{10,000}^{2i/d_{m}}}\right)$$

where pos denotes the time step index and *i* denotes the dimension index. The position-aware representations are processed by a Transformer encoder consisting of L=2 layers [43], each containing a multi-head self-attention mechanism with h=4 heads and a feed-forward network with a hidden dimension of $4d_{m}=256$. Pre-layer normalization is adopted to stabilize training. The multi-head self-attention is computed as:

(8) $$\mathrm{MultiHead}\left(Q,K,V\right)=\mathrm{Concat}\left({\mathrm{head}}_{1},\ldots ,{\mathrm{head}}_{h}\right)W^{O}$$

(9) $${\mathrm{head}}_{j}=\mathrm{Attention}\left(QW_{j}^{Q},KW_{j}^{K},VW_{j}^{V}\right)=\mathrm{softmax}\left(\frac{QW_{j}^{Q}{\left(KW_{j}^{K}\right)}^{⊤}}{\sqrt{d_{k}}}\right)VW_{j}^{V}$$

where $d_{k}=d_{m}/h=16$ is the dimension per head. After the Transformer encoder, temporal mean pooling is applied across all time steps to aggregate the sequence-level information into a fixed-length vector. This vector is then projected through a fully connected layer with batch normalization and ReLU activation to produce the global feature representation $f_{attn}\in R^{d}$.

By attending to all time steps simultaneously, the self-attention branch captures holistic sensor response trends and inter-channel correlations that span the entire measurement duration, providing complementary information to the local features extracted by the CNN branch.

### 3.3. Adaptive Fusion Module

To effectively integrate the local and global feature representations, an adaptive fusion module is introduced that dynamically adjusts the relative contribution of each branch based on the input. Rather than using a fixed weighting scheme, the module employs a learnable gating mechanism that computes a sample-dependent fusion coefficient.

The local and global features are first concatenated and passed through a gating network consisting of two fully connected layers with ReLU activation and a sigmoid output:

(10) $$\alpha =\sigma \left(W_{2}\cdot \mathrm{ReLU}\left(W_{1}\left[f_{cnn};f_{attn}\right]+b_{1}\right)+b_{2}\right)$$

where [·;·] denotes concatenation, α∈(0,1) is a scalar gating coefficient shared across all feature dimensions, and σ(·) is the sigmoid function. The weighted features are then concatenated and projected to form the fused representation:

(11) $$f_{fused}=\mathrm{ReLU}\left(\mathrm{BN}\left(W_{p}\left[\alpha f_{cnn};\left(1-\alpha \right)f_{attn}\right]+b_{p}\right)\right)$$

where $f_{fused}\in R^{d_{f}}$ with $d_{f}=256$. This adaptive mechanism allows the network to emphasize local features for samples where fine-grained patterns are more discriminative, while relying more on global features for samples where long-range dependencies are more informative.

### 3.4. Multi-Granularity Domain Adversarial Learning

To reduce the distribution discrepancy between the source and target domains, MGDA-Net employs a multi-granularity domain adversarial strategy that aligns feature distributions at both the local and global levels simultaneously. Unlike conventional domain adversarial methods that apply a single discriminator to the fused or final-layer features, the proposed approach operates two independent domain discriminators on the CNN branch features and the self-attention branch features, respectively. This design enables finer-grained domain alignment by encouraging cross-domain feature consistency at each feature granularity before fusion.

Each domain discriminator is implemented as an MLP with two hidden layers of 128 and 64 units, ReLU activations, and dropout regularization, followed by a single output unit. The discriminators receive features through a gradient reversal layer (GRL), which acts as an identity function during forward propagation but reverses the gradient sign during backpropagation [44]:

(12) $$\mathrm{GRL}\left(f\right)=f,\;\frac{\partial \mathrm{GRL}(f)}{\partial f}=-\lambda I$$

where λ is the reversal intensity that controls the strength of domain adversarial learning. The domain adversarial loss for each granularity is formulated as a binary cross-entropy loss:

(13) $$L_{\mathrm{domain}}^{\left(g\right)}=-\frac{1}{n_{s}+n_{t}}\left[\sum_{i=1}^{n_{s}}\log\;\left(1-D_{g}\;\left(\mathrm{GRL}(f_{i}^{s})\right)\right)+\sum_{j=1}^{n_{t}}\log D_{g}\;\left(\mathrm{GRL}(f_{j}^{t})\right)\right]$$

where g∈{local,global} denotes the granularity, $D_{g}$ is the corresponding domain discriminator, and $n_{s}$ and $n_{t}$ are the numbers of source and target samples in each mini-batch. The source and target domains are assigned binary domain labels 0 and 1, respectively.

To prevent the domain adversarial signal from destabilizing training in the early stages, a progressive scheduling strategy is adopted for the reversal intensity. The values of $\lambda_{\mathrm{local}}$ and $\lambda_{\mathrm{global}}$ are gradually increased from zero to their respective maximum values following a sigmoid-shaped schedule:

(14) $$\lambda \left(e\right)=\lambda_{\max}\left(\frac{2}{1+\exp(-10\cdot e/E)}-1\right)$$

where *e* is the current epoch and *E* is the total number of training epochs. The maximum reversal intensities are set to $\lambda_{\mathrm{local}}^{\max}=0.2$ and $\lambda_{\mathrm{global}}^{\max}=0.1$, with a higher value assigned to the local branch to encourage stronger alignment of fine-grained local patterns that are more susceptible to domain shift in E-nose data.

### 3.5. Classification with Center Loss Regularization

The fused feature $f_{\mathrm{fused}}$ is fed into an MLP classifier consisting of a fully connected layer with 128 hidden units, batch normalization, ReLU activation, dropout (p=0.5), and a final linear layer that outputs class logits. The classification loss is the cross-entropy loss with label smoothing (ϵ=0.05):

(15) $$L_{cls}=-\frac{1}{N}\sum_{i=1}^{N}\sum_{c=1}^{C}{\tilde{y}}_{i,c}\log\left({\hat{p}}_{i,c}\right)$$

where ${\tilde{y}}_{i,c}=\left(1-\epsilon \right)y_{i,c}+\epsilon /C$ is the smoothed label and ${\hat{p}}_{i,c}$ is the predicted probability for class *c*.

To further enhance intra-class compactness in the feature space, a center loss is incorporated as an auxiliary regularization term [45]:

(16) $$L_{center}=\frac{1}{N}\sum_{i=1}^{N}{∥h_{i}-c_{y_{i}}∥}_{2}^{2}$$

where $h_{i}$ is the hidden feature of sample *i* extracted from the penultimate layer of the classifier, and $c_{y_{i}}$ is the learnable center vector for class $y_{i}$. The center vectors are updated using a dedicated SGD optimizer with a learning rate of 0.5.

### 3.6. Three-Stage Training Strategy

The training of MGDA-Net follows a three-stage progressive strategy that sequentially addresses source-domain learning, cross-domain alignment, and target-domain adaptation.

**Stage 1 (Source-domain pretraining).** In the first stage, the entire network is trained on the labeled source-domain data to learn general-purpose feature representations for tea classification. The training objective combines the classification loss and the center loss:

(17) $$L_{stage1}=L_{cls}+\lambda_{c}L_{center}$$

where $\lambda_{c}=0.01$ is the center loss weight. The model is optimized using the Adam optimizer with a learning rate of $1\times 10^{-3}$ and cosine annealing scheduling for 200 epochs. The model checkpoint with the highest validation accuracy on the source-domain is retained for the next stage.

**Stage 2 (Multi-granularity domain adversarial training).** In the second stage, the feature extractors and domain discriminators are jointly trained to align the source- and target-domain distributions. The source-domain classification loss $L_{cls}^{s}$ is maintained to preserve discriminative capacity, while the domain adversarial losses are introduced at both the local and global feature levels. The total training objective is:

(18) $$L_{stage2}=L_{cls}^{s}+\lambda_{d}\left(L_{domain}^{local}+L_{domain}^{global}\right)$$

where $\lambda_{d}=0.5$ is the domain adversarial weight. Separate optimizers are used for the feature extractors (learning rate $2\times 10^{-4}$) and the domain discriminators (learning rate $1\times 10^{-3}$), with the discriminators trained at a higher learning rate to ensure they provide a strong adversarial signal. This stage is trained for 200 epochs with progressively increasing reversal intensity.

**Stage 3 (Target-domain fine-tuning).** In the third stage, the classifier head is replaced with a new MLP adapted to the target-domain class count, and the center loss module is reinitialized with the corresponding number of target classes. The entire network is then fine-tuned on the labeled target-domain training data. Differential learning rates are applied to different components to preserve the domain-invariant features learned during Stage 2 while allowing the new classifier to adapt quickly. Specifically, the CNN and self-attention branches use a learning rate of $3\times 10^{-5}$, the fusion module uses $5\times 10^{-5}$, and the new classifier uses $1\times 10^{-4}$. The training objective follows the same form as Stage 1 with the center loss regularization. The model is trained for 300 epochs with cosine annealing. To reduce overfitting during target-domain fine-tuning, 20% of the labeled target-domain training samples were randomly split off as a stratified validation subset. The checkpoint with the best validation accuracy was then selected for final evaluation on the held-out target-domain test set.

## 4. Results and Discussion

### 4.1. PCA Visualization of E-Nose Data

**Figure 2:**
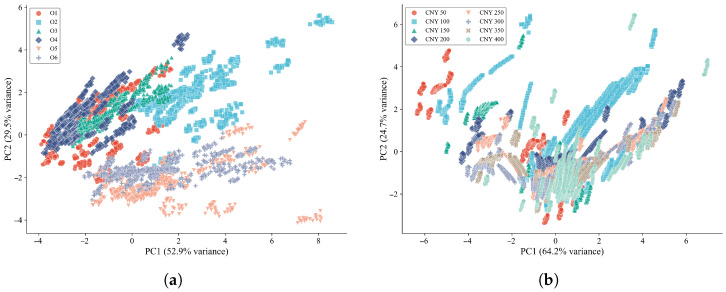
PCA visualization of the steady-state E-nose responses for (**a**) the oolong tea dataset and (**b**) the jasmine tea dataset.

To obtain an initial understanding of the data distribution and class separability in the two target-domain datasets, principal component analysis (PCA) was performed on the steady-state E-nose responses of the oolong tea and jasmine tea samples. Specifically, the responses recorded during the last 20 s of each 120 s measurement cycle were used to represent the stable sensor characteristics. The responses of each sensor over the last 20 s were averaged to obtain a single steady-state value per channel, yielding a 10-dimensional feature vector for each sample. These vectors were standardized to zero mean and unit variance before PCA projection. The two-dimensional PCA results are shown in Figure 2.

For the oolong tea dataset, the first two principal components explained 52.9% and 29.5% of the total variance, respectively, corresponding to a cumulative explained variance of 82.4%. The PCA plot revealed moderate class separability among the six oolong tea categories. In particular, Xupinhao Shuixian and Xupinhao Rougui formed relatively distinct clusters in the lower-right region of the feature space, indicating that their volatile profiles differed markedly from those of the other oolong tea classes. By contrast, substantial overlap was observed among several yancha-related classes, especially Kuidou Shuixian (Premium), Foguoyan Shuixian (A++), and Foguoyan Shuixian (A), which were distributed in closely adjacent regions. These observations suggest that although certain oolong tea categories exhibit distinguishable aroma-response characteristics, fine-grained discrimination among high-end rock tea categories remains challenging when using linear projection alone.

For the jasmine tea dataset, the first two principal components accounted for 64.2% and 24.7% of the total variance, respectively, yielding a cumulative explained variance of 88.9%. Despite the relatively high variance captured by the first two components, the PCA scatter plot showed substantial inter-class overlap among the eight retail price-level categories. Most samples were concentrated in the central region of the projected space, while only a few classes, such as CNY 50 and CNY 100, exhibited partially distinguishable clusters at the periphery. This result indicates that the volatile profiles of jasmine tea samples from different retail price levels are highly similar, and that the differences among adjacent price levels are too subtle to be effectively separated by linear dimensionality reduction.

The PCA results of both datasets demonstrate that conventional linear projection is insufficient for satisfactory separation of the target-domain tea samples, particularly in fine-grained retail price-level classification. These findings highlight the necessity of nonlinear feature learning for robust cross-domain tea classification based on E-nose data.

### 4.2. Comparison with Baseline Methods

To evaluate the effectiveness of the proposed MGDA-Net, eight representative baseline methods were considered, including both classical machine learning classifiers and deep learning-based approaches. The target-only methods comprised three traditional classifiers—support vector machine (SVM), random forest (RF), and k-nearest neighbor (KNN)—and two deep learning models—long short-term memory network (LSTM) and one-dimensional residual network (1D-ResNet). These five methods were trained using only the target-domain training set without access to source-domain data. The classical classifiers were included to quantify how much discrimination can be achieved from target-domain data alone using conventional chemometric features, whereas the transfer-learning baselines were included to assess whether the gains of MGDA-Net arise merely from using source-domain knowledge or specifically from multi-granularity adversarial alignment. LSTM was selected as a widely adopted recurrent architecture for temporal E-nose signal modeling, while 1D-ResNet was included to provide a representative convolutional baseline with residual learning capability. Together, they cover two mainstream deep learning paradigms (recurrent and convolutional) for one-dimensional time-series classification.

For transfer-learning comparison, three widely used domain adaptation methods—deep adaptation network (DAN), domain adversarial neural network (DANN), and conditional domain adversarial network (CDAN)—were implemented under the same source-to-target transfer setting as MGDA-Net, using the three-stage protocol (source pretraining, domain adversarial alignment, and target fine-tuning with classifier replacement). To ensure fairness, all methods used the same day-wise train/test split. Target-only methods were standardized using statistics fitted on the available target-domain training set, whereas transfer-learning methods were standardized using source-domain training statistics because their Stages 1–2 were source-driven. In all cases, preprocessing parameters were estimated without using any test data. For target-only deep models and transfer-learning methods, the same 20% stratified validation subset from the target-domain training portion was used for checkpoint selection during fine-tuning. For SVM, RF, and KNN, hyperparameters were selected on the target-domain training set only. The detailed data usage and preprocessing protocols for each method are summarized in Table 2.

For SVM, a radial basis function (RBF) kernel was used. The regularization parameter *C* and kernel coefficient γ were optimized via 5-fold cross-validation on the target-domain training set, with C∈{0.1,1,10,100} and γ∈{0.001,0.01,0.1,1}. RF was configured with 200 estimators, fully grown trees (no maximum depth constraint), a minimum of two samples per leaf, and the number of features considered at each split set to $\sqrt{p}$, where *p* is the feature dimension. KNN used k=5 neighbors with distance-based weighting and Euclidean distance. LSTM consisted of two bidirectional layers with 128 hidden units, dropout of 0.5 between layers, and a fully connected output layer; it was trained for 500 epochs using the Adam optimizer with a learning rate of $1\times 10^{-3}$ and cosine annealing scheduling. 1D-ResNet comprised an initial convolutional layer followed by three residual stages with 64, 128, and 256 filters, global average pooling, and a fully connected classifier; it was trained under the same optimization setting as LSTM. For DAN, DANN, and CDAN, the backbone feature extractor was a three-layer one-dimensional CNN consistent with the CNN branch of MGDA-Net, and the same three-stage training protocol was applied for fair comparison under heterogeneous source–target label spaces.

To assess robustness to training stochasticity, all methods involving random initialization or stochastic training (RF, LSTM, 1D-ResNet, DAN, DANN, CDAN, and MGDA-Net) were evaluated over 10 independent runs with different random seeds, and the results are reported as mean ± standard deviation. SVM and KNN are deterministic and are therefore reported from a single run.

On the oolong tea dataset (Table 3), MGDA-Net achieved the best performance across all five evaluation metrics, with a mean OA of 99.31 ± 0.69%, a mean F1-macro of 99.30 ± 0.70%, and a mean Kappa coefficient of 0.9917 ± 0.0083 over 10 independent runs. Among the target-only methods, 1D-ResNet achieved the highest mean accuracy (91.60 ± 1.06%), followed by SVM (90.29%) and LSTM (86.18 ± 1.05%). RF and KNN yielded lower accuracies of 85.60 ± 0.46% and 83.27%, respectively. Among the transfer-learning baselines, DAN achieved the highest mean accuracy (88.96 ± 1.05%), slightly outperforming DANN (87.92 ± 1.17%) and CDAN (87.78 ± 1.08%). Compared with the best overall baseline (1D-ResNet), MGDA-Net improved the mean accuracy by 7.71 percentage points. Compared with the best domain adaptation baseline (DAN), the improvement was 10.35 percentage points. The fact that 1D-ResNet outperformed all three standard transfer-learning baselines suggests that single-backbone domain adaptation with only CNN features may not fully exploit the cross-domain structure of the E-nose signals. In contrast, MGDA-Net benefits from multi-granularity feature learning and branch-level adversarial alignment, which together enable more effective knowledge transfer.

On the jasmine tea dataset (Table 4), MGDA-Net again achieved the best results across all metrics, reaching a mean OA of 99.38 ± 0.51%, a mean F1-macro of 99.37 ± 0.51%, and a mean Kappa coefficient of 0.9929 ± 0.0058. This task proved substantially more challenging for all baseline methods, consistent with the PCA in Section 4.1. SVM and KNN achieved accuracies of only 82.35% and 75.27%, respectively, while RF yielded 75.88 ± 0.48%. LSTM achieved 75.56 ± 1.72%, the lowest among the deep learning methods. 1D-ResNet again attained the highest target-only accuracy (87.50 ± 1.52%), confirming that residual learning provides a strong inductive bias for E-nose time-series data even without cross-domain knowledge. Among the domain adaptation baselines, DAN reached the highest mean accuracy (86.94 ± 1.64%), followed by CDAN (86.28 ± 2.31%) and DANN (85.73 ± 1.08%). Compared with the best overall baseline (1D-ResNet), MGDA-Net improved the mean accuracy by 11.88 percentage points. Compared with the best domain adaptation baseline (DAN), the improvement was 12.44 percentage points, representing the largest gain observed across both tasks.

**Figure 3:**
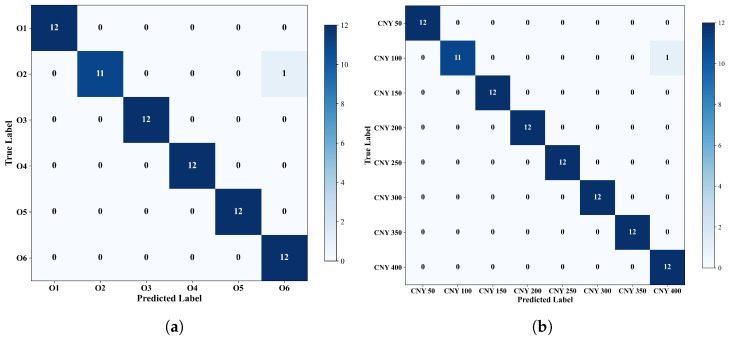
Confusion matrices of MGDA-Net on the target-domain test sets: (**a**) oolong tea task and (**b**) jasmine tea task.

To further examine the class-wise prediction behavior of MGDA-Net, the confusion matrices of a representative run on the oolong tea and jasmine tea test sets are shown in Figure 3.

In the representative run shown in Figure 3, near-perfect classification performance was observed on both target-domain tasks. On the oolong tea test set, only one misclassification occurred among all samples: one Kuidou Shuixian (Grade I) sample was incorrectly predicted as Xupinhao Rougui, while all remaining samples were correctly classified. On the jasmine tea test set, only one error was also observed, where one CNY 100 sample was misclassified as CNY 400. Apart from these isolated cases, no obvious systematic confusion was found among the remaining categories. These representative confusion matrices illustrate that MGDA-Net not only achieves strong overall performance in terms of accuracy, F1-score, and Kappa, but also maintains reliable class-level discrimination on both datasets.

Across both datasets, the results consistently demonstrate the superiority of MGDA-Net over all baseline methods. The improvement was particularly pronounced on the jasmine tea dataset, where fine-grained retail price-level classification posed a greater challenge for conventional classifiers and standard domain adaptation methods. The strong performance and low standard deviation across runs suggest that jointly modeling local and global feature representations, together with branch-level adversarial alignment and adaptive fusion, is both effective and stable for cross-domain tea classification.

### 4.3. Ablation Study

To quantify the contribution of each key component in MGDA-Net, a series of ablation experiments was conducted on both target-domain datasets. Seven ablation variants were designed by removing or modifying one specific component of the full model at a time. Because each ablation variant requires retraining a modified architecture, the results in Table 5 are reported from a representative run under the default experimental setting, whereas the robustness of the full model is quantified separately in Section 4.2.

**Effect of dual-branch feature extraction.** To evaluate the necessity of jointly modeling local and global representations, two single-branch variants were considered. CNN-only retained only the CNN branch, while Attn-only retained only the self-attention branch. On the oolong tea dataset, CNN-only and Attn-only achieved accuracies of 87.15% and 82.64%, respectively, both substantially lower than the full MGDA-Net (98.61%). A similar trend was observed on the jasmine tea dataset, where CNN-only and Attn-only achieved 85.42% and 79.17%, respectively, compared with 98.96% for the full model. Across both datasets, CNN-only consistently outperformed Attn-only, indicating that the CNN branch provides stronger discriminative cues when used alone. However, the large performance gap between either single-branch variant and the full model demonstrates that the two branches are complementary, and that jointly exploiting local and global features is essential for achieving optimal performance.

**Effect of adaptive fusion.** To examine the contribution of the adaptive fusion mechanism, a Concat Fusion variant was implemented by directly concatenating the CNN and self-attention features without learnable reweighting. On the oolong tea dataset, Concat Fusion achieved 84.72% accuracy, which was 13.89 percentage points lower than the full model and even lower than CNN-only (87.15%). On the jasmine tea dataset, Concat Fusion achieved 86.81%, corresponding to a 12.15 percentage point decrease relative to the full model. These results indicate that naive feature concatenation is insufficient for effectively combining heterogeneous branch representations. In contrast, the adaptive fusion module enables dynamic reweighting of local and global features, thereby better exploiting their complementary information.

**Effect of multi-granularity domain adversarial (DA) alignment.** Three variants were designed to investigate the role of domain adaptation and the benefit of feature alignment at different granularities. The w/o DA variant completely removed the domain adversarial training stage, whereas Local-only DA and Global-only DA retained only the local or global domain discriminator, respectively. On the oolong tea dataset, w/o DA achieved 83.68% accuracy, while Local-only DA and Global-only DA achieved 84.38% and 86.11%, respectively. On the jasmine tea dataset, w/o DA yielded 84.03%, Local-only DA yielded 81.94%, and Global-only DA yielded 84.72%. Compared with the full model, removing domain adversarial learning caused substantial performance degradation on both datasets, confirming the importance of cross-domain feature alignment. Moreover, Global-only DA consistently outperformed Local-only DA, suggesting that aligning global contextual representations plays a more important role than aligning local features alone in the present tasks. Nevertheless, neither single-granularity variant matched the full model, demonstrating that local and global alignment provide complementary benefits. Notably, on the jasmine tea dataset, Local-only DA performed even worse than w/o DA, indicating that local-only alignment is insufficient for this more challenging fine-grained classification task.

**Effect of target-domain fine-tuning.** The w/o Fine-tuning variant omitted Stage 3 and directly evaluated the model after Stage 2, without target-domain supervised adaptation of the classifier. This variant produced the lowest performance among all ablation settings, with accuracies of 71.18% on the oolong tea dataset and 73.61% on the jasmine tea dataset. This result is expected because the source-domain and target domains differ not only in data distribution but also in class definition and label space. Without target-domain fine-tuning, the classifier cannot be adequately adapted to the target classification task, even if the learned feature representations have been partially aligned across domains. The marked performance drop further highlights the necessity of Stage 3 for achieving accurate target-domain classification.

Overall, the ablation study demonstrates that each component of MGDA-Net makes a meaningful and non-redundant contribution to the final performance. The dual-branch architecture provides complementary local and global representations, the adaptive fusion module improves feature integration, the multi-granularity adversarial alignment effectively reduces domain discrepancy, and the target-domain fine-tuning stage adapts the classifier to the target task. Removing any of these components leads to a clear reduction in performance, thereby confirming the effectiveness of the proposed design.

### 4.4. Label-Efficiency Analysis

To evaluate the dependence of MGDA-Net on the amount of labeled target-domain data, a series of label-efficiency experiments was conducted. The Stage 1 (source pretraining) and Stage 2 (domain adversarial alignment) models were kept fixed, and only Stage 3 (target fine-tuning) was repeated with progressively reduced proportions of labeled target-domain training data. Specifically, the labeled target-domain training samples were subsampled at ratios of 100%, 80%, 60%, 40%, and 20% using stratified sampling to preserve class balance. For each ratio, 10 independent runs with different random seeds were performed and the results are reported as mean ± standard deviation. The test set (days 9–10) remained unchanged throughout. The results are summarized in Table 6.

Several observations can be drawn from the label-efficiency analysis. First, MGDA-Net exhibits graceful degradation in performance as the proportion of labeled target data decreases. Even when only 40% of the target-domain labels are available (115 samples for oolong tea and 154 samples for jasmine tea), the model still achieves mean accuracies of 88.19% and 87.92%, respectively. These values remain competitive with or superior to several fully supervised baseline methods reported in Table 3 and Table 4. For example, on the oolong tea task, MGDA-Net with 40% labels (88.19%) outperformed LSTM (86.18%), KNN (83.27%), and RF (85.60%), while remaining close to DAN (88.96%). On the jasmine tea task, MGDA-Net with 40% labels (87.92%) slightly outperformed 1D-ResNet (87.50%) and DAN (86.94%).

Second, the performance gap between 100% and 80% labels is relatively small on both tasks, suggesting that the source-domain pretraining and domain-aligned representations already provide a strong initialization, and that the full target-domain training set contains some redundancy for the fine-tuning stage. Third, a more pronounced decline is observed below 40%, particularly for the jasmine tea task, where the standard deviation also increases notably at the 20% level. This indicates that when the number of labeled samples per class becomes very small, the fine-tuning stage becomes more sensitive to the specific samples selected.

These results indicate meaningful label efficiency within the supervised fine-tuning stage of MGDA-Net. At the same time, they should not be interpreted as evidence of unsupervised or few-shot target adaptation, because Stage 3 still relies on labeled target-domain data.

### 4.5. Feature Alignment and Visualization Analysis

To further investigate the effectiveness of MGDA-Net in reducing cross-domain discrepancy and improving feature organization, a series of quantitative and visual analyses was conducted, including multi-kernel maximum mean discrepancy (MMD) and t-distributed stochastic neighbor embedding (t-SNE). Results on both the oolong tea and jasmine tea tasks are discussed below.

#### 4.5.1. Multi-Kernel MMD Analysis

The domain discrepancy between the source-domain (green tea) and the target-domain training set was quantified using multi-kernel MMD with five Gaussian bandwidths at three feature levels, namely the CNN branch output (local feature), the self-attention branch output (global feature), and the fused representation. The MMD values were measured before domain adversarial training (after Stage 1) and after domain adversarial training (after Stage 2).

**Figure 4:**
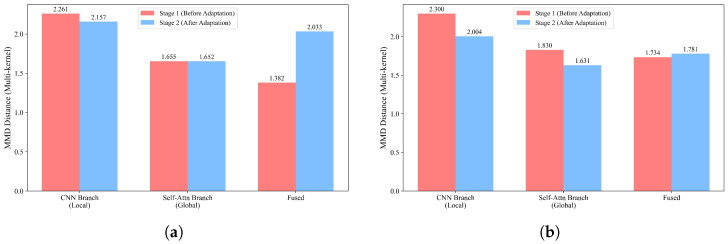
Multi-kernel MMD comparison before and after domain adaptation: (**a**) oolong tea task and (**b**) jasmine tea task.

For the oolong tea task (Figure 4a), domain adversarial training resulted in a modest reduction in the local feature MMD from 2.261 to 2.157, while the global feature MMD remained nearly unchanged (1.655 vs. 1.652). This suggests that the global branch already exhibited a relatively small cross-domain discrepancy before adaptation in the oolong tea task, leaving limited room for further reduction. In contrast, for the jasmine tea task (Figure 4b), domain adversarial training led to clearer reductions in branch-level MMD values: the local feature MMD decreased from 2.300 to 2.004 (−12.9%), and the global feature MMD decreased from 1.830 to 1.631 (−10.9%). These quantitative results are consistent with reduced source–target discrepancy at the branch level after adversarial training. Across both datasets, the global branch exhibited a smaller domain gap than the local branch both before and after adaptation, which is consistent with the ablation results in Section 4.3 showing that Global-only DA outperformed Local-only DA.

It should also be noted that the fused feature MMD did not decrease after Stage 2 on either dataset. For the oolong tea task, the fused MMD increased from 1.382 to 2.033, whereas for the jasmine tea task, it increased slightly from 1.734 to 1.781. This behavior is reasonable because the adversarial alignment in Stage 2 is imposed directly on the branch-level features rather than on the fused representation itself. Meanwhile, the adaptive fusion module dynamically adjusts branch contributions to improve classification-oriented feature integration, which may not always correspond to a monotonic reduction in the fused-space MMD. The final target-domain classification performance is therefore jointly determined by branch-level alignment and subsequent Stage 3 fine-tuning.

#### 4.5.2. t-SNE Visualization

To provide an intuitive view of the learned feature organization, t-SNE was applied to the fused features of the final model after Stage 3 fine-tuning. Two types of visualizations were considered, namely domain-level embeddings showing source and target samples in a shared space, and class-level embeddings showing only the target-domain test samples colored by class label.

**Figure 5:**
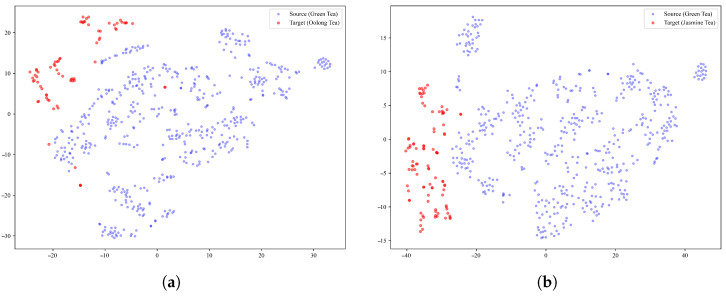
t-SNE visualization of source- and target-domain distributions: (**a**) oolong tea task and (**b**) jasmine tea task.

**Figure 6:**
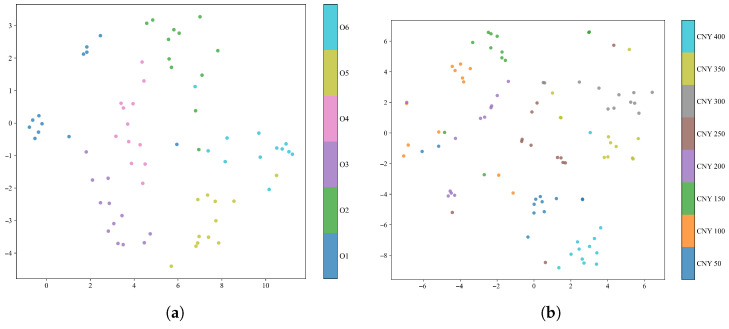
t-SNE visualization of target-domain class distributions: (**a**) oolong tea task and (**b**) jasmine tea task.

As shown in Figure 5, the source-domain and target-domain samples in both tasks were not completely separated into isolated regions at the domain level. Instead, the target samples were distributed in regions adjacent to portions of the source-domain distribution, suggesting partial cross-domain overlap in the learned feature space rather than complete alignment. At the class level, Figure 6 indicates that the target-domain samples formed more structured class-dependent groupings in both tasks. For the oolong tea dataset, six visually distinguishable regions can be observed, whereas the jasmine tea classes remain more closely distributed, which is consistent with the greater difficulty of this fine-grained task. These plots provide descriptive visual evidence of the learned feature organization, but they should not be interpreted as formal proof of discriminative validity or alignment quality.

The quantitative and visual analyses presented above provide complementary evidence consistent with the effectiveness of MGDA-Net. The MMD results suggest that domain adversarial learning reduces source–target discrepancy at the branch level, while the t-SNE visualizations indicate improved feature organization in the projected two-dimensional space. It should be noted that PCA and t-SNE are descriptive dimensionality reduction tools rather than formal tests of discriminative validity or alignment quality; their visualizations should therefore be interpreted as supportive illustrations rather than definitive proof.

## 5. Conclusions

This study proposed MGDA-Net, a multi-granularity domain adversarial framework for cross-domain tea classification using electronic nose signals. By jointly modeling local temporal dynamics and global contextual dependencies and performing branch-level adversarial alignment, MGDA-Net enhances feature transferability from a labeled green tea source-domain to target domains, followed by target-domain fine-tuning to adapt the decision boundary to the target label space.

Comprehensive experiments on two target-domain tasks demonstrated the effectiveness and robustness of MGDA-Net. On the oolong tea dataset (six commercial categories), MGDA-Net achieved a mean accuracy of 99.31 ± 0.69% and a mean Kappa coefficient of 0.9917 ± 0.0083 over 10 independent runs. On the jasmine tea dataset (eight retail price levels), MGDA-Net achieved a mean accuracy of 99.38 ± 0.51% and a mean Kappa coefficient of 0.9929 ± 0.0058. Compared with the respective best overall baseline method (1D-ResNet), MGDA-Net improved the mean accuracy by 7.71 and 11.88 percentage points on the two tasks, respectively. Compared with the best domain adaptation baseline (DAN), the corresponding gains were 10.35 and 12.44 percentage points.

Ablation experiments verified that the dual-branch architecture, adaptive fusion, multi-granularity adversarial alignment, and target-domain fine-tuning each contribute non-redundantly to the overall performance. Label-efficiency experiments further showed that MGDA-Net maintains mean accuracies above 87% even when only 40% of the target-domain labels are used for fine-tuning, indicating that the source-domain knowledge and domain-aligned representations reduce—but do not eliminate—the dependence on labeled target-domain data. Additional distribution and visualization analyses (multi-kernel MMD and t-SNE) provided complementary evidence that the proposed adversarial training improves cross-domain feature consistency.

Several limitations should be acknowledged. First, the target-domain labels used in this study are commercial categories and retail price levels rather than independently validated quality grades based on sensory panel assessment or chemical quality markers. The results therefore demonstrate classification of these specific commercial label sets, and further validation with established quality standards would be needed to extend the conclusions to objective tea quality grading. Second, the method was validated on two target-domain scenarios using a single E-nose instrument type under controlled laboratory conditions; its generalizability to broader tea categories, different instruments, and real-world environmental variations (e.g., temperature and humidity fluctuations) remains to be evaluated. Third, although the label-efficiency experiments show reasonable performance at reduced annotation levels, the current fine-tuning stage still relies on a moderate amount of labeled target data; investigating semi-supervised or few-shot strategies to further reduce labeling requirements is a meaningful direction for future research.

Future work will extend MGDA-Net to additional food and beverage classification tasks, incorporate label-efficient learning strategies, explore model interpretability techniques such as SHapley Additive exPlanations (SHAP) to relate learned features to specific sensor channels and volatile compound profiles, and assess performance under real-world deployment settings with portable electronic nose devices.

## Figures and Tables

**Table 1:** Overview of the three tea datasets used in this study.

Domain	Tea Type	Class Definition	No. of Classes	Samples per Class	Total Samples
Source	Green tea	Different green tea varieties from multiple production regions	12	120	1440
Target 1	Oolong tea	Six commercial categories	6	60	360
Target 2	Jasmine tea	Eight retail price levels (CNY 50–400 per 500 g)	8	60	480

**Table 2:** Summary of data usage and preprocessing for each compared method.

Method	Type	Input Features	Standardization	Training Data (Oolong/Jasmine)
SVM	Target-only	Steady-state (10-dim)	Target training set	288/384
RF	Target-only	Steady-state (10-dim)	Target training set	288/384
KNN	Target-only	Steady-state (10-dim)	Target training set	288/384
LSTM	Target-only	Full time-series (10 × 120)	Target training set	288/384
1D-ResNet	Target-only	Full time-series (10 × 120)	Target training set	288/384
DAN	Transfer	Full time-series (10 × 120)	Source training set	1440 (source) + 288/384 (target)
DANN	Transfer	Full time-series (10 × 120)	Source training set	1440 (source) + 288/384 (target)
CDAN	Transfer	Full time-series (10 × 120)	Source training set	1440 (source) + 288/384 (target)
MGDA-Net	Transfer	Full time-series (10 × 120)	Source training set	1440 (source) + 288/384 (target)

*Note:* Values before “/” refer to the oolong tea task and values after “/” refer to the jasmine tea task. For transfer-learning methods, the source-domain (1440 green tea samples) was used in Stages 1–2, and the target-domain training samples were used in Stage 3. Steady-state features were obtained by averaging the sensor responses over the last 20 s of each 120 s measurement cycle.

**Table 3:** Comparison of classification performance on the oolong tea dataset.

Method	OA	F1-Macro	Kappa	Precision-Macro	Recall-Macro
SVM	0.9029	0.9020	0.8834	0.9142	0.9029
RF	0.8560 ± 0.0046	0.8558 ± 0.0053	0.8272 ± 0.0056	0.8791 ± 0.0060	0.8560 ± 0.0046
KNN	0.8327	0.8256	0.7992	0.8384	0.8327
LSTM	0.8618 ± 0.0105	0.8616 ± 0.0112	0.8421 ± 0.0120	0.8684 ± 0.0107	0.8618 ± 0.0105
1D-ResNet	0.9160 ± 0.0106	0.9153 ± 0.0107	0.8992 ± 0.0127	0.9214 ± 0.0098	0.9160 ± 0.0106
DAN	0.8896 ± 0.0105	0.8899 ± 0.0102	0.8738 ± 0.0120	0.8956 ± 0.0091	0.8896 ± 0.0105
DANN	0.8792 ± 0.0117	0.8798 ± 0.0111	0.8619 ± 0.0134	0.8888 ± 0.0100	0.8792 ± 0.0117
CDAN	0.8778 ± 0.0108	0.8782 ± 0.0114	0.8603 ± 0.0124	0.8849 ± 0.0120	0.8778 ± 0.0108
MGDA-Net	0.9931 ± 0.0069	0.9930 ± 0.0070	0.9917 ± 0.0083	0.9936 ± 0.0064	0.9931 ± 0.0069

**Table 4:** Comparison of classification performance on the jasmine tea dataset.

Method	OA	F1-Macro	Kappa	Precision-Macro	Recall-Macro
SVM	0.8235	0.8206	0.7982	0.8323	0.8235
RF	0.7588 ± 0.0048	0.7511 ± 0.0049	0.7244 ± 0.0055	0.7594 ± 0.0049	0.7588 ± 0.0048
KNN	0.7527	0.7445	0.7174	0.7608	0.7527
LSTM	0.7556 ± 0.0172	0.7560 ± 0.0181	0.7067 ± 0.0207	0.7651 ± 0.0178	0.7556 ± 0.0172
1D-ResNet	0.8750 ± 0.0152	0.8754 ± 0.0149	0.8571 ± 0.0174	0.8875 ± 0.0101	0.8750 ± 0.0152
DAN	0.8694 ± 0.0164	0.8691 ± 0.0163	0.8433 ± 0.0197	0.8725 ± 0.0160	0.8694 ± 0.0164
DANN	0.8573 ± 0.0108	0.8567 ± 0.0111	0.8287 ± 0.0130	0.8617 ± 0.0112	0.8573 ± 0.0108
CDAN	0.8628 ± 0.0231	0.8625 ± 0.0231	0.8354 ± 0.0277	0.8662 ± 0.0222	0.8628 ± 0.0231
MGDA-Net	0.9938 ± 0.0051	0.9937 ± 0.0051	0.9929 ± 0.0058	0.9942 ± 0.0047	0.9938 ± 0.0051

**Table 5:** Ablation study results of MGDA-Net on the oolong tea and jasmine tea datasets. Results are reported from a representative single run; multi-seed robustness of the full model is presented in Table 3 and Table 4.

Dataset	Experiment	OA	F1-Macro	Kappa	Precision-Macro	Recall-Macro
Oolong tea	CNN-only	0.8715	0.8704	0.8458	0.8726	0.8715
	Attn-only	0.8264	0.8264	0.7917	0.8288	0.8264
	Concat Fusion	0.8472	0.8470	0.8167	0.8498	0.8472
	w/o DA	0.8368	0.8361	0.8042	0.8414	0.8368
	Local-only DA	0.8438	0.8435	0.8125	0.8475	0.8438
	Global-only DA	0.8611	0.8601	0.8333	0.8644	0.8611
	w/o Fine-tuning	0.7118	0.7130	0.6542	0.7314	0.7118
	MGDA-Net	0.9861	0.9861	0.9833	0.9872	0.9861
Jasmine tea	CNN-only	0.8542	0.8558	0.8333	0.8699	0.8542
	Attn-only	0.7917	0.7931	0.7619	0.8208	0.7917
	Concat Fusion	0.8681	0.8666	0.8492	0.8730	0.8681
	w/o DA	0.8403	0.8422	0.8175	0.8512	0.8403
	Local-only DA	0.8194	0.8177	0.7937	0.8294	0.8194
	Global-only DA	0.8472	0.8484	0.8254	0.8578	0.8472
	w/o Fine-tuning	0.7361	0.7393	0.6984	0.7701	0.7361
	MGDA-Net	0.9896	0.9896	0.9881	0.9904	0.9896

**Table 6:** Label efficiency results of MGDA-Net on the oolong tea and jasmine tea datasets.

Dataset	Ratio	Labeled	OA	F1-Macro	Kappa
Oolong	100%	288	0.9931 ± 0.0069	0.9930 ± 0.0070	0.9917 ± 0.0083
	80%	230	0.9708 ± 0.0201	0.9707 ± 0.0201	0.9650 ± 0.0241
	60%	173	0.9472 ± 0.0150	0.9473 ± 0.0146	0.9367 ± 0.0180
	40%	115	0.8819 ± 0.0379	0.8829 ± 0.0385	0.8583 ± 0.0455
	20%	58	0.7764 ± 0.0319	0.7754 ± 0.0332	0.7317 ± 0.0383
Jasmine	100%	384	0.9938 ± 0.0051	0.9937 ± 0.0051	0.9929 ± 0.0058
	80%	307	0.9708 ± 0.0153	0.9706 ± 0.0155	0.9667 ± 0.0175
	60%	230	0.9323 ± 0.0248	0.9318 ± 0.0253	0.9226 ± 0.0283
	40%	154	0.8792 ± 0.0229	0.8794 ± 0.0231	0.8619 ± 0.0262
	20%	77	0.7500 ± 0.0395	0.7486 ± 0.0392	0.7143 ± 0.0452

*Note:* For the 100% ratio, the results correspond to the full multi-seed evaluation reported in Table 3 and Table 4. For all ratios, 10 independent runs with different random seeds were performed, varying both the data subsampling and model training stochasticity.

## Data Availability

The code, implementation details, and processed data that support the findings of this study are available from the corresponding author upon reasonable request.

## Abbreviations

The following abbreviations are used in this manuscript:

E-nose	Electronic nose
CNN	Convolutional neural network
MLP	Multilayer perceptron
GRL	Gradient reversal layer
DA	Domain adaptation
MMD	Maximum mean discrepancy
t-SNE	t-distributed stochastic neighbor embedding
PCA	Principal component analysis
SVM	Support vector machine
RF	Random forest
KNN	K-nearest neighbor
LSTM	Long short-term memory
1D-ResNet	One-dimensional residual network
DAN	Deep adaptation network
DANN	Domain adversarial neural network
CDAN	Conditional domain adversarial network
MOS	Metal oxide semiconductor
OA	Overall accuracy
BN	Batch normalization
ReLU	Rectified linear unit
SHAP	SHapley Additive exPlanations
